# Etirinotecan Pegol (NKTR‐102) in Patients With Active Brain Metastases From Lung or Breast Cancer

**DOI:** 10.1002/cnr2.70330

**Published:** 2025-08-28

**Authors:** Seema Nagpal, Kim‐Son Nguyen, Sophie Bertrand, Kristen May Cunanan, Sukhmani K. Padda, Judy Y. Pagtama, Alison Holmes Tisch, Gwen Coffey, Reena P. Thomas, George W. Sledge, Joshua Gruber, Melinda L. Telli, Mark Pegram, Lawrence D. Recht, Heather A. Wakelee, Scott G. Soltys, Suleiman A. Massarweh, Joel W. Neal

**Affiliations:** ^1^ Department of Neurology, Division of Neuro‐Oncology Stanford University Stanford California USA; ^2^ Department of Medicine, Division of Oncology Stanford University Stanford California USA; ^3^ Quantitative Sciences Unit School of Medicine, Stanford University Stanford California USA; ^4^ Fox Chase Cancer Center, Temple University Philadelphia Pennsylvania USA; ^5^ Stanford Health Care Stanford California USA; ^6^ UT Southwestern Medical Center Dallas Texas USA; ^7^ Department of Radiation Oncology Stanford University Stanford California USA

**Keywords:** brain metastases, breast cancer, etirinotecan pegol, NKTR‐102, non‐small cell lung cancer (NSCLC)

## Abstract

**Background:**

Brain metastases are common in patients with lung and breast cancer and are associated with poor outcomes. While there is some intracranial activity with systemic therapies, most chemotherapies are minimally effective. Etirinotecan pegol (EP) is a PEGylated chemotherapy with favorable pharmacokinetics over irinotecan.

**Methods:**

We conducted a phase 2 trial of EP in patients with previously treated metastatic non‐small cell lung cancer (NSCLC, *n* = 12), small cell lung cancer (SCLC, *N* = 3) and breast cancer (MBC, *n* = 12), having progressive brain metastases after brain‐directed radiotherapy (or refusal). The primary endpoint was a 25% or greater disease control rate, defined as CR, PR+SD, in the central nervous system (CNS) at 12 weeks; secondary endpoints included toxicity and systemic disease control.

**Results:**

The CNS control rate at 12 weeks in NSCLC and MBC was 17% (two patients in each cohort) and 0% in SCLC. The median overall progression‐free survival for NSCLC was 2.7 months (95% CI: 1.3, 6.7) and MBC was 1.4 months (95% CI: 1.3, 6.9). The most common adverse events were diarrhea (48%), nausea (48%) and fatigue (26%). Six patient deaths occurred in this study. Dehydration/diarrhea (1) and neutropenic sepsis (3) from study treatment were at least possibly related to these deaths.

**Conclusion:**

This study demonstrates that EP did not meet the threshold of clinical efficacy in patients with refractory CNS metastases from lung or breast cancer.

## 1 Introduction

Brain metastases (BrM) are common in advanced cancers. The development of BrM is associated with decreased quality of life, cognitive changes, and shorter overall survival when compared to patients without BrM [1]. Lung and breast cancers have the highest risk of brain metastases, with 39%–53% of non‐small cell lung adenocarcinoma patients (NSCLC) and 13%–30% of breast cancer patients developing brain metastases over the course of their disease [2]. The mainstays of treatment for BrM are surgical resection and radiotherapy, with a more recent trend towards the use of systemic drugs with central nervous system (CNS) activity. Surgery is often limited by the location and number of metastases. While stereotactic radiosurgery (SRS) can treat multifocal metastases, it does not provide distant CNS control [3]. Whole brain radiotherapy (WBRT) is used for more diffuse metastases and also helps prevent the emergence of new metastases, but it also causes significant acute and chronic toxicities including fatigue, alopecia, gait disturbances, and cognitive decline [4, 5]. Because these local treatments are only temporizing, and may not prevent emerging metastases, effective systemic therapies have the potential to control cancer metastases both systemically and in the brain.

In the last decade, a number of systemic therapies with improved central nervous system (CNS) activity have allowed select patients to avoid or delay radiation or surgery. In particular, patients with epidermal growth factor receptor (EGFR) mutant NSCLC and anaplastic lymphoma kinase (ALK) rearranged NSCLC have access to small molecule tyrosine kinase inhibitors (TKIs) with better CNS response rates than most cytotoxic chemotherapies [6, 7, 8]. For patients with brain metastases from HER2‐positive breast cancer, lapatinib, neratinib, and tucatinib have demonstrated modest activity in brain metastases [9, 10, 11]. Perhaps somewhat surprisingly, given they are large molecules, antibody‐drug conjugates, trastuzumab‐emtansine (T‐DM1) and trastuzumab‐deruxtecan (T‐Dx), also have data supporting single‐agent activity in the CNS. Current academic consensus management guidelines for CNS metastases from breast and lung cancers are constantly being updated [12].

However, the vast majority of patients with lung and breast cancer have tumors that do not harbor mutations amenable to targeting; and even when present, CNS progression is a common and vexing problem. For these patients, the available cytotoxic chemotherapies often have limited efficacy in the brain. Pemetrexed, a small‐molecule anti‐folate used in the treatment of NSCLC, has CNS activity despite a cerebrospinal fluid (CSF) concentration of 2%–3% that of plasma [13]. In a series of 39 patients with NSCLC BrM who received pemetrexed as a secondline or furtherline treatment, 15 patients (38%) had a partial response in the brain, and 12 patients (31%) had stable disease [14]. A randomized phase III study of 105 patients with brain metastases from NSCLC receiving SRS versus upfront platinum and pemetrexed‐ or gemcitabine‐based chemotherapy demonstrated a 37% overall intracranial response rate for patients on the chemotherapy alone arm [15]. For patients with breast cancer, capecitabine alone has reported activity in CNS metastases [16]. Topoisomerase inhibitors like etoposide and irinotecan have also demonstrated CNS activity in both lung and breast cancers, but their modest efficacy coupled with significant toxicities limits their use. There are anecdotal reports of significant CNS disease control by other systemic chemotherapeutics, but most often their activity is limited by drug penetration into the CSF and chemo‐resistance observed late in the disease course [17]. With the relatively dismal CNS response rates from systemic cytotoxic chemotherapies, many patients would benefit from additional non‐targeted chemotherapeutic options that are active against BrM.

Etirinotecan pegol (EP) is a PEGylated chemotherapy formed by steric placement of irinotecan on a four‐armed PEG. PEGylation of drugs may have advantages over the native drug, including reduced enzymatic breakdown and renal clearance, leading to extended drug circulation times. In the Phase 1 study of EP, the elimination *t*_1/2_ for SN‐38, the active metabolite of irinotecan, was 50 days, compared with 12 to 47 h for the native drug [18]. A subsequent phase II study of EP in patients with recurrent glioblastoma after bevacizumab demonstrated both safety and activity in the CNS, with 3 out of 17 patients having a partial response, 2 of which were durable beyond 18 months [19]. In the phase III BEACON study, 852 patients with previously treated locally recurrent or metastatic breast cancer were randomized to EP versus a regimen of physician's choice (i.e., choice of seven standard regimens) [20]. The subset of patients with brain metastases who received EP rather than physician's best choice had a significant overall survival benefit (10.0 months v. 4.8 months, HR = 0.51, *p* < 0.01) [21]. The combination of extended drug exposure and preliminary evidence of activity in the CNS made EP a promising candidate for the treatment of refractory brain metastases from lung and breast cancers.

In this study, we prospectively tested the CNS efficacy of EP in active, refractory brain metastases in patients with NSCLC, small cell lung cancer (SCLC), and metastatic breast cancer (MBC).

## 2 Methods

### 2.1 Eligibility and Treatment

The Stanford University Institutional Review Board approved this study and it was conducted in accordance with the principles of Good Clinical Practice and applicable clinical trial regulations. All participants provided written informed consent prior to participation in the study. The trial was registered with ClinicalTrials.gov (NCT02312622).

This was a 3‐arm, prospective, non‐randomized trial enrolling adults (> 18 years old) with active, progressive brain metastases into three cohorts: NSCLC (*n* = 12), SCLC (*n* = 3), or MBC (*n* = 12). Patients with ECOG performance status (PS) 0–2 were enrolled at the Stanford Cancer Institute (Stanford, CA, USA) from February 2015 to June 2018. Patients were required to have an ECOG (PS) of 0–2 and had received at least one line of prior systemic (chemotherapy or targeted) treatment for metastatic disease, or adjuvant chemotherapy within 6 months of disease recurrence. Patients with NSCLC had standard of care molecular testing; those with an EGFR mutation or ALK rearrangement had to progress after at least one standard TKI; those with MBC had standard of care ER/PR/HER2 testing and at least one taxane‐based regimen.

Patients were required to receive at least one prior CNS‐directed therapy (surgery, SRS, or WBRT) or opt out of offered treatment. All patients had measurable CNS disease defined as: (1) at least one CNS target lesion 10 mm or greater in longest diameter or (2) at least one CNS target lesion measuring 5–9 mm in longest diameter, plus one or two additional CNS target lesions measuring 3 mm or greater in longest diameter, for which the sum of the longest diameters of these lesions is equal to or greater than 10 mm. Previously irradiated CNS lesions were permitted to be target lesions provided there was evidence of growth not believed to be due to radiation necrosis or edema. All patients were required to have adequate bone marrow, liver, and renal function. Patients with persistent diarrhea who required chronic or frequent use of anti‐diarrheal medications were not eligible.

Patients received etirinotecan pegol 145 mg/m^2^ IV as monotherapy once every 21 days until disease progression or unacceptable toxicity. Systemic response was assessed with a contrast CT, and CNS response was assessed by MRI every 6 weeks for as long as patients remained on study. Response was measured by RECIST 1.1 criteria, with response confirmation not required in the CNS or systemically. For patients with BrM that required summation to meet entry criteria, the sum of growth or shrinkage for those lesions had to meet RECIST criteria for response or progression.

### 2.2 Trial Design and Statistics

This was a single‐stage phase II design with the primary end point of CNS disease control rate (SD, PR, or CR in the CNS) by RECIST 1.1 criteria at 12 weeks. In the NSCLC and MBC cohorts, three or more patients out of 12 with CNS disease control at 12 weeks would reject the null hypothesis that the disease control rate is less than or equal to 5%. The sample size provided 81% power with a one‐sided significance level of 5%, with the calculation based on binomial probabilities and assuming an alternative of 33% for the CNS disease control rate. The study also included an exploratory, observational group of three patients with SCLC. Progression‐free survival (PFS) and overall survival (OS) curves were estimated using the Kaplan–Meier method, calculated from the start of the drug to the time of event or censoring. Patients who withdrew for clinical decline believed to be disease‐related by the investigator were considered to have progressive disease. Patients who came off the study for toxicity without evidence of systemic progressive disease were censored at the off‐study date. Summary statistics were used for the secondary endpoints of toxicity and systemic disease control.

## 3 Results

### 3.1 Demographics

Twenty‐seven eligible patients were enrolled and received their first dose of etirinotecan pegol between January 2015 and June 2018 (Table 1). Eight patients (three with NSCLC and five with MBC) were not evaluable for radiographic response because MRI, CT, or both were not performed.

**TABLE 1:** Patient and disease characteristics by cohort.

Patient and disease characteristic	All (*n* = 27)	NSCLC (*n* = 12)	SCLC (*n* = 3)	MBC (*n* = 12)
Median age (range)	51 (32–80)	54 (35–79)	58 (51–80)	47 (32–65)
Ethnicity				
Hispanic		0	0	1
Non‐Hispanic White		3	3	9
Asian		9	0	2
Lung Adenocarcinoma	12	12	NA	NA
EGFR		6 (50%)		
KRAS		1 (8%)		
ERBB2		1 (8%)		
ROS1		1 (8%)		
NRAS		1 (8%)		
None		2 (17%)		
Breast Adenocarcinoma	12	NA	NA	12
Triple positive				1 (8%)
Triple negative				5 (42%)
ER+/PR+/HER2−				4 (33%)
ER−/PR−/HER2+				1 (8%)
ER+/PR−/HER2 unknown				1 (8%)
Sex (F:M)	22:5	8:4	2:1	12:0
Median prior systemic therapy (range)	3 (1–11)	2.5 (1–8)	1 (1–3)	4 (1–11)
Prior neurosurgical resection	5 (19%)	2 (17%)	0	3 (25%)
Prior WBRT	12 (44%)	3 (25%)	1 (33%)	8 (67%)
Prior radiosurgery	18 (67%)	9 (75%)	0	9 (75%)

The median age was 51 years (range 32–80). All patients in the MBC cohort were women, while 8 of 12 (67%) NSCLC patients were women. Eight patients in the breast cancer cohort had received prior WBRT, while only three patients in the NSCLC cancer cohort had prior WBRT. The median number of prior lines of systemic therapy was 2.5 (range 1–8) in the NSCLC cohort and 4 (range 1–11) in the MBC cohort. Patients across all cohorts received a median of 2 doses of EP range (1–15).

### 3.2 Safety

The most common treatment emergent adverse events (AEs) of EP were diarrhea (48% of patients), nausea (48%), and fatigue (26%). All grade 1–2 treatment related AEs occurring in more than 10% of patients and all grade 3–5 events regardless of attribution are shown in Table 2. Dose delays were required in four patients (15%) and dose reductions in 3 patients (11%). One patient in the MBC cohort was treated past technical progression for clinical benefit.

**TABLE 2:** Adverse events, related in > 10% of patients or grade 3 or higher, regardless of attribution.

Adverse event	Grade 1–2 (probably, possibly, definitely related occurring in > 10% of patients) *n* (%)	Grade 3–4 (regardless of attribution) *n* (%)	Grade 5 (regardless of attribution) *n* (%)
Diarrhea	13 (48%)	4 (15%)	0
Nausea	12 (44%)	2 (7%)	0
Fatigue	7 (26%)	1 (4%)	0
Blurred vision	5 (19%)	0	0
Photophobia	5 (19%)	0	0
Vomiting	4 (15%)	2 (7%)	0
Dyspepsia	3 (11%)	0	0
Anorexia	3 (11%)	0	0
Low ANC	2 (7%)	1 (4%)	0
Sepsis	0	1 (4%)	3 (11%)
Dehydration	0	1 (4%)	1 (4%)
Non‐cardiac chest pain	0	2 (7%)	0
Thromboembolic event	0	2 (7%)	0
Febrile neutropenia	0	2 (7%)	0
Seizure	0	2 (7%)	0
Pleural effusion	0	2 (7%)	0
Colitis	0	1 (4%)	0
Hypocalcemia	0	1 (4%)	0
Hypophosphatemia	0	1 (4%)	0
Hypovolemia	0	1 (4%)	0
Anemia	0	1 (4%)	0
Pancytopenia	0	1 (4%)	0
Cardiogenic shock	0	1 (4%)	0
Pericardial tamponade	0	1 (4%)	0
Shock liver	0	1 (4%)	0
Upper respiratory infection	0	1 (4%)	0
Fall	0	1 (4%)	0
Vertebral fracture	0	1 (4%)	0
Back pain	0	1 (4%)	0
Dysphasia	0	1 (4%)	0
Cerebral edema	0	1 (4%)	0
Stroke	0	1 (4%)	0
Pelvic pain	0	1 (4%)	0
Hypertension	0	1 (4%)	0
Hypotension	0	1 (4%)	0
Disease progression	0	0	1 (4%)
Respiratory failure	0	0	1 (4%)

Six patients died while on study: two patients died from progressive disease, three patients died from sepsis, and one patient died of diarrhea‐related dehydration. The sepsis events all occurred in the setting of neutropenia refractory to growth factor support and antibiotics during hospitalization. The death due to dehydration occurred during hospitalization with aggressive supportive care. These events of sepsis and diarrhea were attributed at least possibly to study drug, with underlying comorbidities from cancer also contributing.

### 3.3 Efficacy

The CNS disease control rate at 12 weeks was 17% (two patients each) in the breast and NSCLC cohorts; thus, the primary endpoint was not met in either. The overall (systemic+CNS) disease control rate in both the NSCLC and MBC cohorts was also 17% (two patients each). All patients had a median PFS of 1.4 months (95% Confidence Interval (CI): 1.3, 2.7) and OS of 7.1 months (95% CI: 3.4, 12.3).

**FIGURE 1:**
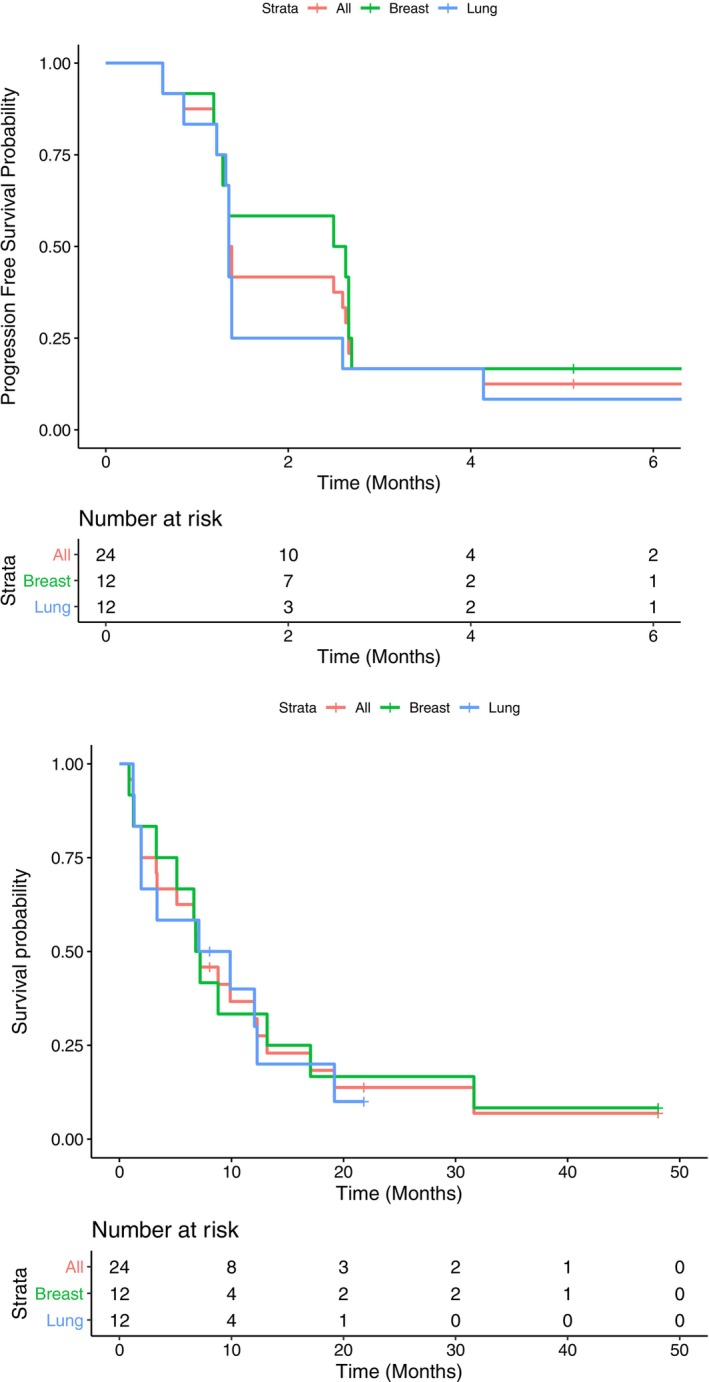
Progression free survival and overall survival by disease and overall. Upper panel: The median PFS for the breast and lung cohorts was 1.4 months (95% CI 1.3, 6.7) and 2.6 months (95% CI 1.3, 6.7), respectively. The overall median PFS was 1.4 months (95% CI 1.4, 2.7). Lower panel: The median OS for the MBC and NSCLC cohorts was 8.5 months (95% CI 1.9, 21.8) and 7.0 months (95% CI 5.1, 48.0), respectively. The overall median OS was 7.2 months (95% CI 5.1, 13.2).

In the NSCLC cohort, PFS and OS were 2.6 months (95% CI: 1.3, 6.7) and 7.0 months (95% CI: 5.1, 48), respectively (Figure 1). Three patients had PR in the CNS (unconfirmed on subsequent scans); two of these patients also had a systemic response, while one had stable systemic disease. For six patients with EGFR mutant NSCLC, median OS was 11 months (95% CI: 6.6, 48).

In the MBC cohort, the PFS and OS were 1.4 (95% CI: 1.3, 6.9) and 8.5 (95% CI: 1.9, 21.8) months, respectively (Figure 1). One patient had a PR in the brain (confirmed on a subsequent scan), two had stable disease in the CNS but experienced systemic disease progression. Five patients had CNS progression with stable systemic disease.

**FIGURE 2:**
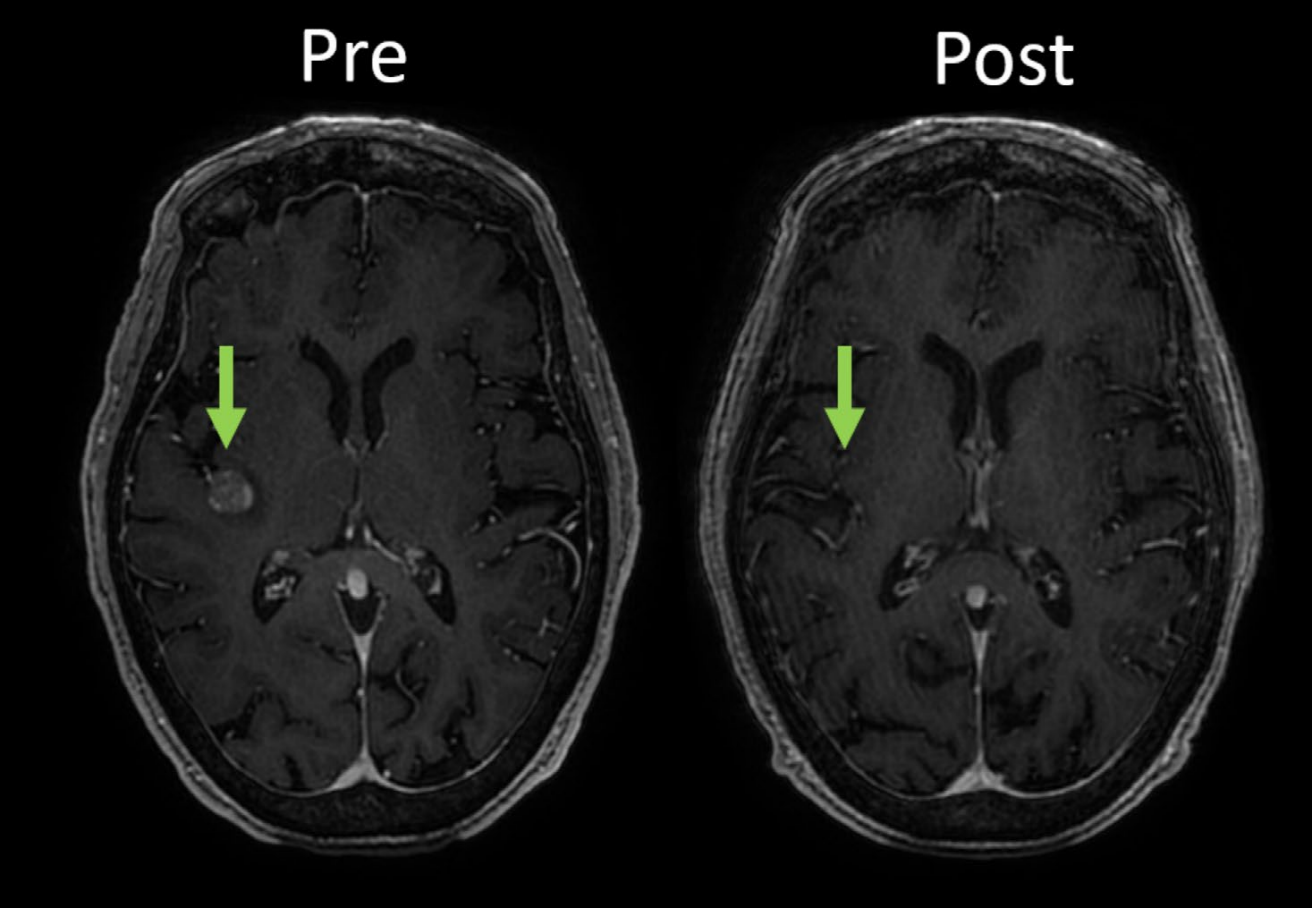
CNS efficacy in patient with SCLC. MRI of axial T1 post gadolinium images shown. Left panel, pre‐treatment. Right panel, 6 weeks post EP therapy.

In the SCLC cohort, two of three patients had CNS responses on the 6‐week MRI, but they developed subsequent sepsis and died before response confirmation. Images from one of these patients showing a response are presented (Figure 2).

## 4 Discussion

In this pilot study, etirinotecan pegol did not meet the target threshold of 25% CNS response rate in active, refractory BrM. Limited activity was observed in patients with breast cancer and non‐small cell lung cancer. This modest activity was in the setting of clinically relevant toxicity, including deaths possibly related to the drug.

The observed CNS and overall disease control rate of 17% is modest when compared with targeted agents having CNS activity in breast and lung cancers, but it is in line with studies of chemotherapy in patients with heavily pretreated brain metastases. For example, the combination of capecitabine and lapatinib produced an objective response rate of 66% in a study of patients with previously untreated brain metastases [9], but a meta‐analysis of 12 studies using lapatinib in a broader array of patients, most of whom had received three or more prior lines of treatment, demonstrated a pooled CNS response rate of 21% [22]. Interestingly, EP did produce an objective response in the two SCLC patients who had not received prior brain radiation, though these patients did not have confirmatory scans due to subsequent adverse events. This suggests that this agent is indeed active in the CNS. However, in other patients, its efficacy may be limited by penetration in the blood–brain barrier, as well as intrinsic chemoresistance of metastatic disease to the CNS, which is common in heavily pretreated populations [23, 24]. It is also unclear how tumor heterogeneity and the tumor microenvironment, which are important factors in the development of brain metastases, may affect response to this agent [25, 26]. Regarding the systemic activity of EP, after completion of our study a phase II study by Aggarwal in NSCLC was reported with low response rates, with two patients of 40 achieving a partial response (ORR 5%) and median PFS was 2.3 months, lower than generally observed from single‐agent docetaxel in the second‐line setting.

Most large Phase 3 studies that include patients with BrM require patients either to have asymptomatic, treatment naïve BrM, or to have stable treated BrM at the time of enrollment. Frequently, the final publications from these studies do not distinguish between these two when reporting outcomes, or do not report CNS outcomes at all. Notably, the ATTAIN study of EP did not include patients with active or treatment naïve BrM, but included a similar population to the patients on the BEACON study: those with stable, previously treated BrM. The DESTINY‐Lung01 trial of T‐Dx, which included patients with asymptomatic BrM, did not report CNS PFS or RR in the paper or supplemental material. The variability in BrM patient populations and lack of uniform data make direct comparison of this study to contemporary studies challenging. However, the median OS in our study is similar to the median OS of 5.5 months reported in a group of breast and lung cancer patients who received cisplatin and temozolomide for previously treated brain metastases [27]. While the authors did not present overall survival by tumor type, they noted a higher CNS response rate in breast cancer patients, with 6 of 15 (40%) patients achieving PR, while the NSCLC cohort had 2 responses in 11 patients (18%). Combining all cohorts in our study, the median OS was 7.1 months. In patients with NSCLC, the median OS was 7.0 months, and despite a lower radiographic response rate, the median OS was 8.5 months for patients with breast cancer. While we looked for patterns based on molecular subtypes, the five triple negative breast cancer patients and six patients with targetable EGFR mutations did not demonstrate any clear patterns of response.

The toxicity observed in this study was clinically significant, with a total of six deaths, four of which were not exclusively related to disease progression. The underlying metastatic tumor burden and patient condition were likely strong contributing factors to the deaths observed on study, even those attributed to treatment. The 15% rate of Grade 3 or 4 diarrhea in this study was comparable to the phase III BEACON study (10%); although, in a small study of heavily pretreated high‐grade glioma patients at our institution, there was only one incident of Grade 3 diarrhea [19, 28]. All patients on our study had been mandated to follow an anti‐diarrheal protocol with a low threshold for loperamide, counseling for immediate reporting to the study team, and weekly telephone check‐ins to assess diarrhea, but this was not completely effective as the patient that died from dehydration developed diarrhea and vomiting yet did not seek medical care for a week. The BEACON study also reported that 10% of patients experienced grade 3–4 neutropenia related events. In this study, there were three (11%) treatment‐related deaths due to sepsis, and two of these were in patients with SCLC in the setting of neutropenia. It is possible that prior myelo‐suppressive cytotoxic chemotherapy used for SCLC treatment (e.g., platinum‐etoposide) potentiated the subsequent neutropenic toxicity effects of EP. Additionally, the long half‐life of EP (50 days) may have led to prolonged drug exposure requiring proactive and extended supportive care measures. Growth factor support was not mandated for cycle 1, but was permitted in subsequent cycles, and potentially in future investigations could be administered routinely to prevent toxicity. Overall, the toxicities encountered in this study may limit the routine use of EP in this population with heavily pretreated disease. Future studies with this therapy might also consider limiting inclusion performance status to ECOG 0‐1.

Our study had several limitations, including the relatively small number of patients in the study and the inclusion of < 1 cm brain metastases as target lesions. Regarding CNS response criteria, this study was written using modified RECIST criteria and opened to enrollment just prior to the publication of the Response Assessment in Neuro‐Oncology Brain Metastases (RANO‐BM) groups' initial response criteria in 2015 [29]. Notably, RECIST and RANO‐BM require a 1 cm minimum dimension for measurable disease. However, in our experience, it is increasingly uncommon for patients to have untreated lesions that are larger than 1 cm as brain metastases greater than 2–3 mm often undergo radiosurgery. Therefore, we developed customized criteria for measurable disease in this trial to increase the population of eligible patients: patients with multiple smaller brain metastases (3–5 mm) were able to enroll, provided they had at least one metastasis of 5 mm or larger and a number of other metastases 3 mm or larger, adding to at least 10 mm. Notably, approximately one third of the patients on this study would not have qualified for enrollment using RANO‐BM or RECIST criteria. Given that the PFS and OS in this study are comparable to historical studies, it is unlikely that these criteria significantly altered the outcome of the study.

In conclusion, our study demonstrated limited activity of etrinotecan pegol in patients with lung or breast cancer and active brain metastases. While evidence of CNS responses was observed, the study did not meet the primary endpoint of 25% disease control rate for either the NSCLC or breast cancer cohorts, and the toxicities encountered were clinically significant. We, therefore, do not recommend pursuing a randomized clinical trial using this agent in pre‐treated or progressive brain metastasis. However, there remains a large, unmet need for anti‐cancer agents in patients with brain metastases from a variety of tumor types, particularly as advances in systemic therapy often have sub‐optimal activity in the CNS.

## Author Contributions

**Seema Nagpal:** conceptualization (equal), formal analysis (equal), methodology (equal), writing – original draft (equal), writing – review and editing (equal). **Kim‐Son Nguyen:** conceptualization (equal), methodology (equal), writing – original draft (equal), writing – review and editing (equal). **Sophie Bertrand:** data curation (equal), methodology (equal), writing – original draft (equal), writing – review and editing (equal). **Kristen May Cunanan:** formal analysis (equal). **Sukhmani K. Padda:** data curation (equal), writing – review and editing (equal). **Judy Y. Pagtama:** data curation (equal), writing – review and editing (equal). **Alison Holmes Tisch:** data curation (equal), writing – review and editing (equal). **Gwen Coffey:** data curation (equal), writing – review and editing (equal). **Reena P. Thomas:** data curation (equal), writing – review and editing (equal). **George W. Sledge Jr:** data curation (equal), writing – review and editing (equal). **Joshua Gruber:** data curation (equal), writing – review and editing (equal). **Melinda L. Telli:** data curation (equal), writing – review and editing (equal). **Mark Pegram:** data curation (equal), writing – review and editing (equal). **Heather A. Wakelee:** data curation (equal), writing – review and editing (equal). **Scott G. Soltys:** conceptualization (equal), data curation (equal), writing – review and editing (equal). **Suleiman A. Massarweh:** conceptualization (equal), methodology (equal), writing – review and editing (equal). **Joel W. Neal:** conceptualization (equal), writing – original draft (equal), writing – review and editing (equal).

## Conflicts of Interest

Consulting/Advisory Role: Mirati, Pyramid Biosciences, Seattle Genetics, Novocure, Nektar Therapeutics, AstraZeneca, Sanfi Genzyme, Amgen, Bayer, Regeneron, Mirati Therapeutics, Johnson & Johnson, Rayze Biotech, Jazz Pharma, Nanobiotix, Genentech, Blueprint Medicines, G1 Therapeutics, Sharma Therapeutics, Merck, Immunomedics, Genentech/Roche, G1 Therapeutics, Natera, Pfizer, OncoSec, Blueprint Medicines, Guardant Health, Novartis, AstraZeneca, Sanofi, Reflexion Medical, Gilead Sciences, Genentech/Roche, Pfizer, Seattle Genetics, Lilly, AstraZeneca, Novartis, IOBiotech, Mirati Therapeutics, BMS (uncompensated), Genentech/Roche (uncompensated), Merck (uncompensated), AstraZeneca (uncompensated), Accuray Inc., Novocure Inc., AstraZeneca, Genentech/Roche, Exelixis, Takeda Pharmaceuticals, Eli Lilly and Company, Amgen, Iovance Biotherapeutics, Blueprint Pharmaceuticals, Regeneron Pharmaceuticals, Natera, Sanofi/Regeneron, D2G Oncology, Surface Oncology, Turning Point Therapeutics, Mirati Therapeutics, Gilead Sciences, AbbVie, Summit Therapeutics, Novartis, Novocure, Janssen Oncology, Anheart Therapeutics. Research funding (to institution): Pyramid Biosciences, ABM Therapeutics, Novocure, Nektar Therapeutics, Nektar Therapeutics, Inovio Pharmaceuticals, PharmAbcine, Oncoceutics, Novocure, BPG Bio, Servier, Incyte, Biocept, VBI Vaccines, EpicentRx, Bayer, Boehringer Ingelheim, Bioalta, Takeda Pharmaceuticals, Bristol‐Meyers Squibb, Chimerix, Hummingbird Biosciences, Curis, Novartis, PharmaMar, AbbVie, Calithera Biosciences, Genentech, Medivation, OncoSec, Vertex, Biothera, Tesaro, Pfizer, EMD Serono, Bayer, AstraZeneca/Medimmune, Bayer, BMS, Genentech/Roche Helsinn, Merck, SeaGen, Xcovery, Novocure Inc., Genentech/Roche, Merck, Novartis, Boehringer Ingelheim, Exelixis, Nektar Therapeutics, Takeda Pharmaceuticals, Adaptimmune, GSK, Janssen, AbbVie, Nuvalent. Honoraria: Genentech/Roche, Pfizer, Seattle Genetics, Lilly. Speaker Honoraria: Accuray Inc. Zap Surgical Inc.

## Data Availability

Data available on request due to privacy/ethical restrictions.
